# Thrombus formation induced by laser in a mouse model

**DOI:** 10.3892/etm.2014.1677

**Published:** 2014-04-14

**Authors:** PABLO PÉREZ, MARCELO ALARCÓN, EDUARDO FUENTES, IVÁN PALOMO

**Affiliations:** 1 Department of Clinical Biochemistry and Immunohematology, Faculty of Health Sciences, Interdisciplinary Excellence Research Program on Healthy Aging (PIEI-ES), University of Talca, Talca, Maule 3460000, Chile; 2 Centro de Estudios en Alimentos Procesados (CEAP), CONICYT-Regional, Talca, Maule R09I2001, Chile

**Keywords:** thrombosis formation, rose bengal, platelet

## Abstract

Animal models are used for the development of techniques and/or models that aid the study of thrombosis pathophysiology. The aim of the present study was to modify the technique of *in vivo* thrombosis induction to make it more accessible. BALB/c mice were intraperitoneally anesthetized with 0.4 ml 2,2,2-tribromoethanol (266.6 mg/kg) and xylazine (13.3 mg/kg), whilst maintaining stable blood pressure and temperature. Through abdominal surgery, the mesentery was identified and isolated for the visualization of the arteries. A simple epifluorescence magnifier was used to detect the presence of thrombi. The results obtained indicate that using rose bengal at concentrations of 25 and 50 mg/kg and a laser power of 5 mW, thrombus formation occurred. In addition, formation of the thrombus occurred ~30 min following induction and the thrombus had a total area of 4,878.3 μm^2^, which caused total occlusion of the mesenteric artery. For visualization, platelets were labeled with calcein acetyloxymethyl ester for 1 h, which resulted in improved observation of thrombus formation in real time. Therefore, this technique may be used to perform *in vivo* studies simply and at low cost, and is suitable for use in a variety of studies of thrombosis.

## Introduction

Cardiovascular diseases (CVDs) result in >19 million mortalities annually, and coronary heart disease contributes significantly to these mortalities. Individuals with the disease may appear healthy, yet succumb to CVD suddenly without prior symptoms (1).

In the majority of cases, the development and progression of CVD is characterized by the interaction of atherosclerotic lesion and thrombus formation processes, and platelet participation is pivotal in these processes (2,3). Following an atheromatous plaque rupture, platelets adhere, secrete their granule contents, aggregate and initiate thrombus formation (3). Activation is a dynamic process that may lead to intermittent or permanent obstruction of blood flow, resulting in ischemic tissue injury and organ dysfunction (4).

The inhibition of platelet function has been used for a long time as a method to prevent ischemic complications at late stages of the atherosclerotic process; ischemic complications are a leading cause of cardiovascular morbidity and mortality (5,6). Animal models are used to provide a cost effective means for the development of techniques and/or models that aid the study of thrombosis pathophysiology. Previous studies of animal models have used a variety of techniques to induce thrombosis, including the application of ferric chloride (a compound that produces endothelial damage, causing an injury from the adventitia to the intima that induces a loss of endothelial integrity) (7), stasis (based on stopping the blood flow and thereby causing damage to the blood vessel, which leads to a hypercoagulable state) (7), ultrasound (which also induces damage in the endothelium) (8) and laser treatment (9–13). Laser irradiation, which causes a directed photosensitization reaction, has been reported as a method of forming thrombi by inducing endothelial damage in a murine model (9). The reactive oxygen species, which are generated by the interaction of rose bengal and the laser, play a relevant role in the model since tissue damage contributes to pathological environments in thrombosis formation (14–16).

The present study describes a model of laser-induced thrombosis that has been modified to be relatively inexpensive yet remain effective. The main objective of the study was to develop a low cost *in vivo* thrombosis model that is able to provide results comparable with those of more sophisticated and expensive systems.

## Materials and methods

### Reagents

Rose bengal, calcein acetyloxymethyl ester (AM), 2,2,2-tribromoethanol, 2-methyl-2-butanol and dimethylsulfoxide were obtained from Sigma-Aldrich (St. Louis, MO, USA).

### Animal model

Male BALB/c mice aged 8–10 weeks (weight, 25–35 g) were bred and housed in groups of six mice per cage. The mice were fed a pelleted basal diet (CRF-1; Oriental Yeast Co., Ltd., Tokyo, Japan) and provided with free access to drinking water. Mice were maintained in the animal house facilities at University of Talca (Talca, Chile) under standard conditions of relative humidity (50±10%), temperature (23±2°C) and light (12/12 h light/dark cycle), according to the Institutional Animal Care Guidelines. The experimental procedures were reviewed and approved by the Animal Care and Use Committee at University of Talca.

### In vivo murine model of thrombosis

BALB/c mice were anesthetized (0.4 ml) using a combination of tribromoethanol (270 mg/kg) and xylazine (13 mg/kg). Mice were placed in the supine position with the hind legs clamped and stretched to achieve tension in the abdominal area. The mesentery was exposed by performing a central incision in the abdomen, permitting the visualization of thrombus development in the mesenteric vessels.

In order to induce thrombosis in the shortest possible time, rose bengal was administered. Intravenous injections of various concentrations (5, 10, 25 and 50 mg/kg) of rose bengal were administered into the tail vein, following heating of the tail with a lamp for 3 min. Thus, thrombosis was induced in the mesenteric arteries following illumination of the exposed mesenteric area with a 5-mW green light laser (532 nm; O/E Land, Inc., Montréal, QC, Canada). Images were captured every 10 min using a zoom stereomicroscope (SMZ800; Nikon, Tokyo, Japan). Color images were received by a camera (MF-TV; Nikon, Tokyo Japan) that was connected to a computer system (INFINITY 1; Lumenera Corp., Ottawa, ON, Canada). Rectal temperatures were similar and within the physiological range among all experimental animals throughout the experimental period. Blood flow was monitored for 60 min and stable occlusion was defined as a blood flow of 0 ml/min for 3 min. Area measurement of the thrombus was performed with the software ImageJ (National Institutes of Health, Bethesda, MD, USA).

### Preparation of the human platelet suspensions

Venous blood samples were obtained from two young healthy volunteers who had previously provided informed consent. The samples were collected in 3.2% citrate tubes (9:1 v/v) by phlebotomy with a vacuum tube system (Becton Dickinson, Franklin Lakes, NJ, USA). The experimental procedures were authorized by the Ethics Committee of the University of Talca and were conducted in accordance with the Declaration of Helsinki (approved by the 18th World Medical Assembly in Helsinki, Finland, 1964). Tubes were centrifuged (DCS-16 Centrifugal Presvac RV; Presvac, Buenos Aires, Argentina) at 240 × g for 10 min to obtain platelet-rich plasma (PRP). The PRP was adjusted to 2×10^11^ platelets/l with platelet-poor plasma obtained by centrifugation of the original tubes at 650 × g for 10 min. Washed platelets, at a concentration of 2×10^11^ platelets/l, were prepared in Tyrode-HEPES buffer containing 50 ng/ml prostaglandin E1 (PGE_1_) and 1 mmol/l ethylenediamine-N,N,N′,N′-tetraacetic acid (EDTA; pH 7.4). To avoid platelet activation, 50 ng/ml PGE_1_ was added to the PRP prior to centrifugation at 750 × g for 10 min. Platelet counts were performed in a hematologic counter (Advia 60 Hematology System; Bayer Corporation & Diagnostics, Tarrytown, NY, USA).

### Dynamics of platelet thrombus formation

Washed platelets (2×10^11^ platelets/l) were labeled with 4 μmol/l calcein AM for 1 h in the dark. Then, 0.2 ml platelets were intravenously injected into the tail vein of the mice. Next, it was necessary to exteriorize and isolate the mesentery, securing a stable platform to achieve maximum tension. An epifluorescence microscope (Thermo Fisher Scientific, Inc., New York, NY, USA) was used to identify the blood vessels. Then, using a magnification of ×63, the injury induction process was initiated. Thrombus formation was monitored over a 60-min period as aforementioned.

### Statistical analysis

Data were analyzed using SPSS version 17.0 software (SPSS, Inc., Chicago, IL, USA) and expressed as mean ± standard error of the mean. Three or more independent experiments were performed for the various assays. Differences among groups were analyzed with the unpaired t-test and by one-way analysis of variance using Tukey’s post-hoc test. P<0.05 was considered to indicate a statistically significant difference.

## Results

### Effect of rose bengal on laser-induced thrombosis

**Figure 1:**
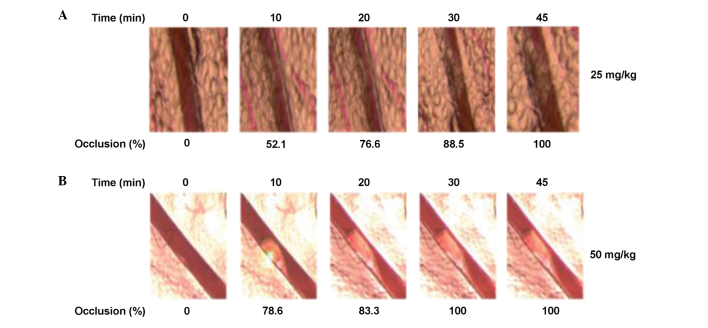
Real-time thrombus formation with (A) 25 and (B) 50 mg/kg rose bengal. Magnification, ×63.

The effects of various concentrations of rose bengal on the stimulation of thrombosis formation are shown in Table I. Laser irradiation with 5 and 10 mg/kg concentrations of rose bengal was ineffective in initiating thrombosis formation in the mesenteric arteries. By contrast, at concentrations of 25 and 50 mg/kg, thrombus formation (total occlusion) occurred at ~45 and ~30 min, respectively (Fig. 1). Additionally was observed that the presence of the laser and rose bengal together was required for the induction of thrombosis, and that either alone did not induce thrombosis (data not shown).

**Figure 2:**
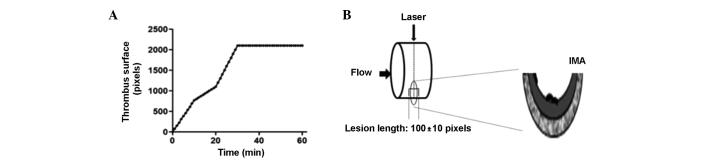
Thrombus formation induced with a laser and 50 mg/kg rose bengal. (A) Typical tracing representing the development of the thrombus with time, following deep laser injury. (B) Schematic representation of the lesion along the artery (left) and in a transversal section at the centre of the lesion (right).

**Figure 3:**
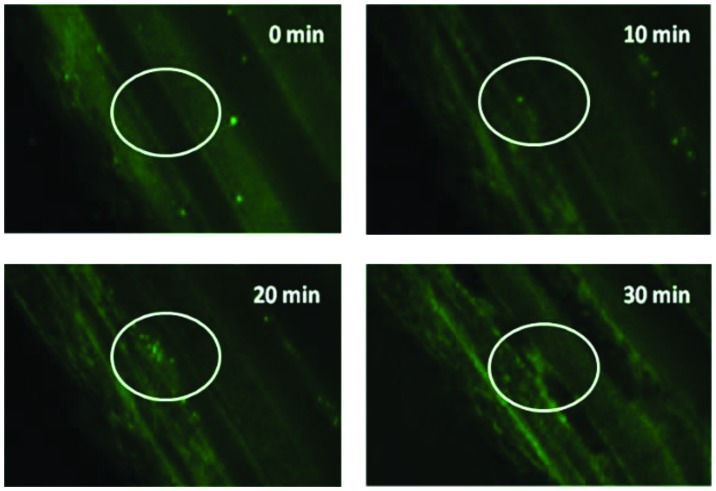
*In vivo* thrombus formation following laser irradiation at 50 mg/kg rose bengal. Platelets labeled with calcein AM (green).

Fig. 2A shows thrombus formation over time at a concentration of 50 mg/kg. Following laser irradiation, the thrombus formed within 30 min, causing total occlusion of the vessels. During the 60 min of monitoring, the formation of a stable thrombus and blood vessel occlusion was observed. The thrombus was 4,878.3 pixels in size and occluded the blood vessel by 100% at 30 min (n=6). Fig. 2B schematically shows the incidence of the laser beam causing localized damage to the endothelium. Fig. 3 shows *in vivo* thrombus formation following irradiation with 50 mg/kg rose bengal and fluorescence detection using calcein-labeled platelets.

## Discussion

The present study establishes a simple system using a rose bengal concentration of 50 mg/kg and a laser power of 5 mW that is effective for use in studies of thrombosis. The results obtained also indicate that increasing the concentration of rose bengal, whilst maintaining the same laser power (5 mW), decreases the time taken for thrombus formation in the mesenteric arteries. Previous studies have described techniques for imaging thrombi formed by photochemical action on arteries and/or arterioles in a murine model (17–19). Thrombus formation following laser irradiation in the mesenteric arteries has been evaluated *in vivo* in our laboratory using an adaptation of the system used by Sigler *et al* (10).

Experimental models of thrombosis are essential for the study of the complex factors involved in thrombus formation; however, each model has advantages and limitations (20). The majority of models are based on the initiation of thrombosis by injury to the endothelium, which exposes subendothelial components and subsequently leads to the development of thrombosis (21).

Fukuoka *et al* (9), showed that thrombus formation using a laser (9.8 mW; 532 nm) directed at the pial artery in C57BL/6 mice induced thrombosis. In the present study, an adaptation of the method was used to induce thrombosis formation in BALB/c mice using a laser with a power of 5 mW and a wavelength of 532 nm. Rose bengal was administered to the mice, which were then irradiated with green light from the laser. This process generates singlet oxygen and superoxide anions in the irradiated site, leading to endothelial cell damage at the site of injury (16). During the induction of thrombus formation using rose bengal, there is no extravasation damage to the vasculature; therefore, damage occurs only at the site of irradiation (16,22).

Przyklenk *et al* (20) demonstrated that the use of 20, 25 and 50 mg/kg rose bengal and a laser power of 0.34 mW did not result in thrombus formation. However, thrombus formation did occur when the laser power was increased to 0.84 mW with 25 mg/kg rose bengal (22). In the present study, it was shown that a specific laser power of 5 mW and concentrations of 5 and 10 mg/kg rose bengal did not result in thrombus formation in the mesenteric arteries. However, increasing the concentration of rose bengal to 25 and 50 mg/kg did result in thrombus formation. In this context, a major difference is that the laser in the present study had a greater power, producing greater endothelial damage, thereby enhancing the induction of thrombus formation. The results demonstrate that laser power, the site of induction and the concentration of rose bengal are important factors in thrombus formation. The images captured allow the extraction of substantial and important information, which improves the description of the results. The key to this technique is the use of fluorophores that are activated at longer wavelengths, allowing thrombus detection (17). This type of technique allows the study of thrombi formation in real time, yielding excellent results. However, the costs are extremely high, as expensive microscopes and accessories are required. Therefore, these techniques are used only in laboratories that are well-equipped and well-funded (17,23,24). In the present study, the technique was modified to make it less expensive and remained highly effective for the analysis of thrombi formed in real time. Visualization of the clot was performed using platelets previously labeled with calcein AM and an epifluorescence microscope was used for thrombus imaging. Therefore, the modification of this technique allows the study of a wide range of functions, including the study of thrombosis and antithrombotic agents *in vivo*.

In conclusion, thrombosis was successfully induced using the modified mouse model of *in vivo* thrombosis described in the present study. The effect of a given concentration of rose bengal, a specific laser power and the integration of labeled platelets allowed the visualization and evaluation of thrombi production in a mouse model. Therefore, this *in vivo* system of thrombosis may be useful in future studies due to its low cost and high reproducibility. In addition, modification of the technique is likely to enable its use in various types of *in vivo* studies, including studies that demonstrate the antithrombotic effects of any compound and/or molecule. Therefore, this modified mouse model may be a critical tool for the study of CVD.

## Figures and Tables

**Table I:** Photochemically induced thrombosis with various concentrations of rose bengal in mice.

Rose bengal (mg/kg)	Laser fluence (mW/mm^2^)	Mice (n)	Outcome
5	255	3	No thrombosis
10	255	3	No thrombosis
25	255	3	Total occlusion
50	255	3	Total occlusion
